# Recent Updates of Transarterial Chemoembolilzation in Hepatocellular Carcinoma

**DOI:** 10.3390/ijms21218165

**Published:** 2020-10-31

**Authors:** Young Chang, Soung Won Jeong, Jae Young Jang, Yong Jae Kim

**Affiliations:** 1 Department of Internal Medicine, Digestive Disease Center, Institute for Digestive Research, Soonchunhyang University College of Medicine, Seoul 04401, Korea; chyoung@schmc.ac.kr (Y.C.); jyjang@schmc.ac.kr (J.Y.J.); 2 Department of Radiology, Soonchunhyang University College of Medicine, Seoul 04401, Korea; rtwodtwo@schmc.ac.kr

**Keywords:** transarterial chemoembolization, hepatocellular carcinoma, refractoriness, combination therapy

## Abstract

Transarterial chemoembolization (TACE) is a standard treatment for intermediate-stage hepatocellular carcinoma (HCC). In this review, we summarize recent updates on the use of TACE for HCC. TACE can be performed using two techniques; conventional TACE (cTACE) and drug-eluting beads using TACE (DEB-TACE). The anti-tumor effect of the two has been reported to be similar; however, DEB-TACE carries a higher risk of hepatic artery and biliary injuries and a relatively lower risk of post-procedural pain than cTACE. TACE can be used for early stage HCC if other curative treatments are not feasible or as a neoadjuvant treatment before liver transplantation. TACE can also be considered for selected patients with limited portal vein thrombosis and preserved liver function. When deciding to repeat TACE, the ART (Assessment for Retreatment with TACE) score and ABCR (AFP, BCLC, Child-Pugh, and Response) score can guide the decision process, and TACE refractoriness needs to be considered. Studies on the combination therapy of TACE with other treatment modalities, such as local ablation, radiation therapy, or systemic therapy, have been actively conducted and are still ongoing. Recently, new prognostic models, including analysis of the neutrophil-lymphocyte ratio, radiomics, and deep learning, have been developed to help predict survival after TACE.

## 1. Introduction

Primary liver cancer is the sixth most commonly diagnosed cancer and the fourth leading cause of cancer mortality worldwide. Hepatocellular carcinoma (HCC) accounts for 75% to 95% of all primary liver cancer cases [1]. HCC-related mortality continues to increase despite the overall declining trends in cancer incidence and death rates. Because of its global disease burden and poor prognosis, HCC is considered a major global health problem.

Prognosis of HCC patients is highly heterogeneous and depends on various factors such as tumor burden, baseline liver function, cancer-related symptoms represented by performance status, and treatment allocation [2,3]. According to the Barcelona Clinic Liver Cancer (BCLC) staging system, which has been commonly used in clinical practice and endorsed by international guidelines, transarterial chemoembolization (TACE) is the treatment of choice for intermediate-stage HCC, including unresectable multinodular HCC without extrahepatic spread. The BCLC system additionally recommends that TACE should be used when other recommended treatments are not feasible or unsuccessful in the early stages of HCC. In Asian countries, TACE tends to be more broadly recommended for various clinical situations [4]. Although the clinical situations in which TACE is indicated differ slightly depending on the various staging systems, TACE is a well-established treatment for intermediate-stage HCC [3,5]. In this review, we summarize recent data from studies regarding TACE, including various techniques, evidence for combination strategies, and potential new indications. We hope to thus provide updated guidance for treatment decisions.

## 2. Conventional TACE and Drug-eluting Beads Using TACE

There are two TACE techniques; conventional TACE (cTACE), which uses lipiodol, and drug-eluting beads (DEBs) which use TACE (DEB-TACE).

### 2.1. Conventional TACE

Conventional (c)TACE involves intra-arterial injection of cytotoxic agents such as doxorubicin or cisplatin emulsified in the oil-based radio-opaque agent, lipiodol. This is followed by an intra-arterial injection of embolic agents such as a gelatin sponge. In cTACE, lipiodol delivers cytotoxic agents directly to the tumor itself and causes embolization of the tumor microcirculation. Furthermore, intratumoral retention of lipiodol can be detected on post-procedural imaging, which enables the prediction of the treatment response. The superiority of cTACE over the best supportive care for intermediate-stage HCC has been confirmed by many randomized controlled trials (RCTs), meta-analyses, and systematic reviews [6,7,8]. Based on these data, cTACE has been considered a standard treatment for intermediate-stage HCC with the highest grade of recommendation [9,10].

### 2.2. Drug-Eluting Beads Using TACE

DEBs, which are non-resorbable embolic microspheres capable of releasing drugs, were developed to achieve sustained targeted release of cytotoxic agents with concomitant tumor embolic effects. DEBs of various sizes from manufacturers have been developed and tested for their efficacy when used in DEB-TACE. Although there has been no direct comparative study on the different DEB devices available thus far, beads 100–300 μm in size have been most commonly used, and smaller DEBs, such as those with sizes of 70–150 μm or 30–60 μm, have been recently evaluated for their efficacy [11,12,13,14].

## 3. Comparison of the Efficacy and Safety of cTACE and DEB-TACE

### 3.1. Efficacy

Several prospective studies and meta-analyses have compared the efficacy of cTACE and DEB-TACE and showed no significant differences in tumor response, time to progression, or overall survival (OS) between the two [15,16,17]. In the PRECISION V multicenter RCT phase II study, DEB-TACE failed to show a better tumor response than that shown by cTACE [15]. There was no significant difference in tumor response on magnetic resonance imaging (MRI) at the primary endpoint, six months after the procedure (*p* = 0.11). The PRECISION ITALIA STUDY GROUP phase III trial also failed to show a statistical difference in tumor response, time to progression, and survival between cTACE and DEB-TACE [16]. This trial initially planned to enroll 214 patients, but was stopped due to futility after 177 patients were enrolled; 88 in the cTACE group, and 89 in the DEB-TACE group. The one-year survival rate was 86.2% in the DEB-TACE group and 83.5% in the cTACE group, while the 2-year survival rates were 56.8% and 55.4%, respectively (*p* = 0.949). A recent meta-analysis involving four RCTs and eight observational studies confirmed the non-superiority of DEB-TACE over cTACE in terms of tumor response achieved and the one-, two-, and three-year survival rates [17].

### 3.2. Safety

In terms of safety issues, the most frequent adverse event after TACE is the post-embolization syndrome characterized by abdominal pain, fever, and ileus. One systematic review reported that post-embolization syndrome and a transient increase in liver enzymes occurred in 47.7% and 52% of the patients who underwent cTACE, respectively [8]. DEB-TACE was expected to be associated with a lower rate of adverse events, including post-embolization syndrome. However, unexpectedly, DEB-TACE failed to show superiority over cTACE with respect to safety endpoints. In the PRECISION V trial, the incidence of serious adverse events within 30 days after the procedure were 20.4% in the DEB-TACE group and 19.4% in the cTACE group (*p* = 0.86) [15]. One meta-analysis also found no significant difference in adverse events between the groups (*p* = 0.36) [17]. Overall, there was no significant difference in the adverse event rates between DEB-TACE and cTACE, except for the rate of postprocedural pain, which was less frequently associated with DEB-TACE [16]. Moreover, liver/biliary injuries, including biloma and liver infarction, were independently associated with DEB-TACE (OR, 6.63; *p* < 0.001), and DEB-TACE was linked to significantly more frequent procedure-related locoregional complications such as biliary injury (OR, 4.53; *p* < 0.001) and global hepatic damage (OR, 3.13; *p* < 0.001) than cTACE [18,19]. The incidence of hepatic arterial damage, which is associated with OS, was significantly higher after DEB-TACE than after cTACE (OR, 3.13, *p* = 0.005) [20]. In addition, it has recently been reported that the frequency of arterio-portal shunt formation was significantly higher in Child-Pugh class A patients who underwent DEB-TACE [21].

Taken together, the evidence is still insufficient to show that DEB-TACE is superior to cTACE in terms of efficacy and safety.

### 3.3. Balloon-Occluded TACE

A novel technique called balloon-occluded TACE (B-TACE), first reported by Irie et al., has recently been developed in Japan [22]. B-TACE is defined as the infusion of a chemotherapeutic emulsion with lipiodol followed by gelatin particles under feeding artery occlusion by a microballoon catheter [23]. The occlusion of feeding arteries results in a dense accumulation of the chemotherapeutic emulsion with lipiodol in the target nodules. Several studies have reported that the therapeutic efficacy of B-TACE is superior to that of conventional TACE [24,25,26]. However, these studies were retrospective and involved small sample sizes. Well-designed RCTs comparing B-TACE to conventional TACE or DEB-TACE are thus warranted.

## 4. Application of TACE Outside of Intermediate-Stage HCC

### 4.1. Early-Stage HCC

TACE is primarily recommended for patients with intermediate-stage disease. For patients with early stage HCC, liver transplantation, surgical resection, or local ablation are generally recommended as curative treatments [9,10]. However, some patients are not good surgical candidates due to several clinical factors such as old age, hepatic dysfunction, and severe comorbidities [27]. Furthermore, the shortage of liver donors is a major limitation of liver transplantation [28]. Although local ablation is considered a safer alternative to surgery in these situations, it is also not suitable for tumors with a subcapsular or dome location or in tumors located near the main bile duct, large vessels, or intestinal loops [29]. Patients who cannot benefit from curative treatment, despite earlier stage disease, could be good candidates for TACE. This treatment stage-migration strategy is well established and recommended by international guidelines [9,10,30]. Several studies have reported a high response rate and good outcomes after TACE in patients with early stage HCC for whom curative treatment is not feasible owing to various clinical factors [31,32,33]. TACE can be used as neoadjuvant therapy before liver transplantation (LT). In such cases, TACE serves as a downstaging therapy, allowing a patient to become suitable for LT, or as a bridge therapy while the patient is on the waiting list [9,34]. Several studies have demonstrated that TACE decreases the dropout rate from the waiting list of LT to 3–13%, especially when the expected waiting time for LT exceeds six months [34,35,36]. Moreover, response to preoperative TACE has been confirmed to correlate with post-transplant tumor recurrence and OS [37,38,39].

### 4.2. Advanced-staGe HCC

Advanced stage HCC (BCLC stage C) is characterized by cancer-related symptoms with/without vascular invasion or extrahepatic metastasis with preserved liver function and performance status. Sorafenib is the treatment of choice for advanced HCC, and lenvatinib has also been recommended as a first-line systemic treatment after its non-inferiority to sorafenib was demonstrated [40,41]. More recently, the combination of atezolizumab and bevacizumab resulted in better OS and progression-free survival (PFS) than sorafenib, and has been approved by the U.S. Food and Drug Administration for advanced HCC patients who have not received prior systemic therapy [42]. For sorafenib-experienced patients, regorafenib, cabozantinib, ranucirumab, and nivolumab can be used as second- or third-line treatments [43,44,45,46]. However, the population with advanced-stage disease is heterogeneous because the extent of portal vein tumor thrombosis (PVTT) and extrahepatic spread is not considered. Approximately 20–30% of the newly diagnosed HCC patients have PVTT; this proportion increases up to 42% in patients without HCC surveillance [47,48], and all of these patients are considered to have advanced-stage disease. The extent of PVTT can vary, ranging from the involvement of the small segmental branch to the main trunk and beyond, and it has been repeatedly reported that the extent of PVTT, not just the presence of PVTT, is an important determinant of survival [49,50]. Nevertheless, according to most treatment guidelines, the presence of PVTT severely restricts treatment options, regardless of the extent of PVTT. Systemic chemotherapy with sorafenib or lenvatinib other than local treatment is the only proven standard treatment in such cases [51,52,53].

Despite the stipulated guidelines, TACE was implemented as the first-line treatment in nearly 50% of the cases of BCLC-C stage HCC in an international large-scale longitudinal cohort study that reflected real-world clinical practice [54]. The rationale for the application of TACE in HCC with PVTT is that collateral vessel formation around the portal vein allows for the preservation of liver function, which in turn makes TACE possible in selected cases with segmental or subsegmental PVTT [55]. The survival benefit afforded by TACE over that afforded by best supportive care has been demonstrated in various studies [56,57,58].

To summarize, TACE is recommended not only for intermediate-stage HCC, but also for early stage HCC as a stage-migration strategy and neoadjuvant treatment. Although TACE is not recommended as a standard therapy in most cases of advanced-stage HCC, it can be considered as a treatment option in selected patients with segmental PVTT and preserved liver function. Further study comparing TACE and systemic therapy as first-line treatment in selected patients is necessary.

## 5. TACE Failure/Refractoriness: Repeat or Stop?

### 5.1. Scoring Systems Used between TACE Sessions

In patients treated with TACE, the usual oncological parameters to determine the treatment response are not always valid, as local tumor progression can generally benefit from repeated TACE sessions. Therefore, deciding whether to repeat or stop TACE is difficult and often subjective. The Assessment for Retreatment with TACE (ART) score [59] and the ABCR (AFP, BCLC, Child-Pugh, and Response) score [60] were developed to identify patients who may benefit from repeated TACE (Table 1). The ART score is based on the existence of a serum aspartate aminotransferase (AST) increase > 25%, an increase in the Child-Pugh score, and the absence of radiological tumor response after the first TACE. This score ranges from 0 to 8, with an ART score ≥ 2.5 indicating that repeated TACE may not be effective [59,61]. The ABCR score included baseline AFP ≥ 200 ng/mL, baseline BCLC stage, an increase in the Child-Pugh score by ≥2 points, and radiological tumor response after the first TACE. An ABCR score ≥ 4 indicates that patients may not benefit from further TACE sessions [60].

However, while the ART and ABCR scores are useful scoring systems that can be easily calculated and help predict treatment response, their predictive value has not been well validated. Several studies have reported that the ART score failed to predict overall survival in patients who received repeated TACE [62,63,64]. The ABCR score as well as ART score failed to show sufficient prognostic ability to guide the decision-making process regarding subsequent TACE [65]. The fact that ART and ABCR scores are not reflected in the current treatment guidelines suggests that neither score has been sufficiently validated. Clinicians should, therefore, make clinical decisions after the careful assessment of an individual patient’s various clinical features, rather than relying entirely on these scoring systems.

### 5.2. Discontinuing Rules of TACE

There have been several studies regarding the discontinuation of TACE. In an RCT by Lo et al., repeated TACE was discontinued in patients with poor hepatic function, severe adverse effects, or major progressive disease [66]. Poor hepatic function was defined as the presentation of hepatic encephalopathy, uncontrolled ascites, variceal bleeding, serum bilirubin > 3 mg/dL, serum albumin < 2.8 g/dL, or prothrombin time prolongation > 4 s over the control. Progressive disease was defined based on an increased tumor size and AFP of more than 25%. In a systematic review by Llovet et al., TACE was discontinued when a patient presented Child-Pugh class C, gastrointestinal bleeding, hepatic encephalopathy, uncontrolled ascites, and progressive disease, including vascular invasion or extrahepatic spread [7]. Untreatable tumor progression after TACE involves massive liver involvement, vascular invasion, extrahepatic involvement, and minor tumor progression with deterioration of hepatic function or performance status [67]. It is clear that patients with this condition will not benefit from repeated TACE.

### 5.3. TACE Failure/Refractoriness

The concept of TACE refractoriness was first proposed by the Japan Society of Hepatology (JSH) [68]. The JSH criteria defined TACE failure/refractoriness based on the following four indications: progression of intrahepatic lesions, continuous elevation of tumor markers, vascular invasion, and extrahepatic spread [69]. The progression of intrahepatic lesions was further defined as two or more consecutive insufficient responses of the treated tumor presenting viable lesions > 50%, or two or more consecutive increases in tumor numbers (Table 2). There are still various opinions on the criteria of TACE refractoriness, and physicians often have to make subjective judgments based on heterogeneous clinical presentations [70]. It is clear that the survival of patients with unresectable HCC varies greatly, depending on the time at which TACE refractoriness is recognized and the treatment plan is switched [71].

Although several attempts have been made to discover the molecular pathways responsible for TACE refractoriness, the underlying molecular mechanisms have yet to be fully elucidated. Jun et al. reported that c-MET, associated with resistance to anticancer therapies in various malignancies, was upregulated in HCC after TACE, and that upregulated c-MET was associated with TACE refractoriness [72]. A recent study on the p53 pathway, the second most commonly defective pathway in HCC [73], reported that p53 mutation was independently related to TACE failure/refractoriness via the mitogen-activated protein kinase and apoptosis pathways [74]. In addition, SIRT7 was found to suppress the transcriptional activity of p53 by deacetylation, thus contributing to HCC progression [75]. SIRT7 expression is highly associated with TACE refractoriness and poor survival [75]. These molecular pathways may provide evidence for the identification of patients who will benefit from TACE, and may also represent potential novel therapeutic targets for treating TACE-refractory HCC.

### 5.4. Impact of Repeated TACE on Liver Function

It has been well demonstrated that intrahepatic tumor control and hepatic reserve are the most important prognostic factors in patients with HCC, even with extrahepatic metastasis [76,77]. One of the key considerations when treating HCC is to preserve liver function as much as possible with effective intrahepatic tumor control. Several studies have shown that patients treated with TACE, especially those treated with less selective or repeated TACE, have impaired liver function [78,79]. In patients with refractory disease, repeated TACE may lead to a deterioration in liver function, resulting in missed opportunities for other systemic therapies, and consequent reduced overall survival. A timely switch to an appropriate systemic therapy before deterioration of liver function is critical for safe follow-up treatment.

## 6. Combination Treatment with TACE

### 6.1. TACE with Radiofrequency Ablation

An RCT in patients with a solitary recurrent HCC lesion < 5 cm in diameter demonstrated that sequential therapy with TACE followed by radiofrequency ablation (RFA) significantly improved OS compared to the therapy with RFA alone [80]. The one-, three-, and five-year survival rates were 94%, 69%, and 46% for the combination therapy, respectively, and 82%, 47%, and 36% for RFA alone (*p* = 0.037). In addition, sequential combination therapy resulted in significantly longer recurrence-free survival in patients with tumors larger than 3 cm in diameter [80]. Another RCT demonstrated the superiority of the TACE-RFA combination therapy over RFA alone in patients with HCC lesions with a diameter of less than 7 cm in both OS (HR = 0.525; *p* = 0.002) and recurrence-free survival (HR = 0.575; *p* = 0.009) [81]. For small HCC lesions (2–3 cm in diameter), there was no significant difference in long-term therapeutic outcomes between TACE combined with RFA and surgical resection, which implies that the TACE/RFA combination therapy could be an alternative treatment for patients with a single small HCC lesion for whom surgical resection is unsuitable [82]. Two meta-analyses reported that TACE combined with RFA was more effective than RFA alone, especially for intermediate- and large-sized HCC, and in younger patients with HCC [83,84].

### 6.2. TACE with Radiation Therapy

There has been mounting evidence regarding the efficacy of combination therapy of TACE and radiation therapy (RT) for treating patients with intermediate-stage HCC, as well as advanced-stage HCC with PVTT [85,86,87]. According to an extensive meta-analysis involving 11 RCTs and 14 non-RCTs, treatment with TACE plus RT resulted in significantly improved response and survival rates compared to TACE alone in patients with unresectable HCC [88]. In addition, TACE plus RT has shown a promising response rate and OS among HCC patients with macrovascular invasion in several observational studies [89,90,91]. Based on these observational studies, a well-designed RCT was conducted, which demonstrated that first-line treatment with TACE plus RT was well tolerated and improved various treatment outcomes compared to those associated with sorafenib treatment among advanced-stage HCC patients with macrovascular invasion [92]. This research provides a new treatment paradigm to treat patients with locally advanced HCC using a combination of TACE and RT.

### 6.3. TACE with Systemic Therapy

The anticancer mechanisms of TACE involve a tumor embolic effect leading to tissue necrosis, in addition to the local delivery of cytotoxic agents. TACE causes tissue hypoxia that results in the upregulation of vascular endothelial growth factor (VEGF), which may lead to tumor revascularization and local recurrence. In this regard, the combination of antiangiogenic agents with TACE was expected to inhibit the revascularizing action of upregulated VEGF induced by TACE. Accordingly, the combination of TACE with anti-angiogenic agents may delay tumor progression or recurrence, and thus improve OS.

Several attempts have been made to combine TACE with other systemic agents whose main mechanism of action is anti-angiogenesis (Table 3): The SPACE and TACE 2 trials compared TACE plus sorafenib and TACE alone [93,94], while the BRISK-TA study did the same with brivanib, and the ORIENTAL study with orantinib [95,96]. Despite the plausible rationale for the combination strategy, all four RCTs evaluating the combination of TACE with systemic agents failed to show any clinical benefit compared to TACE alone. Kudo et al. presented several reasons for these repeated negative trials [97]. They suggested that the duration of the study was too short to evaluate OS as a primary endpoint. In addition, post-trial treatments after progression likely affect OS, making it difficult to evaluate treatment outcomes using OS. The definition of time to progression or PFS also needs to be more standardized, and tailored to the specifics of TACE treatment. Based on the lessons from the previous trials, Kudo et al. demonstrated positive results of the TACTICS trial, a randomized phase II trial comparing TACE plus sorafenib with TACE alone [98]. The use of TACE plus sorafenib resulted in a major improvement in PFS: 25.2 months in the TACE plus sorafenib group versus 13.5 months in the TACE alone group (*p* = 0.006). The improved outcomes observed in the TACTICS trial can be explained by the differences in the study protocol compared to those of the previous trials. The most distinctive difference is that new intrahepatic lesions were not regarded as progressive disease because they do not imply treatment failure based on the natural tumor biology of HCC. Progression in this trial was defined as untreatable (unTACEable) progression; e.g., > 25% of intrahepatic tumor progression, deterioration of liver function to Child-Pugh class C after TACE, macrovascular invasion, or extrahepatic spread. Treatment was continued until unTACEable progression, TACE refractoriness, or unacceptable toxicity. Sorafenib was started two–three weeks prior to the first TACE in this trial. As a result, patients in the TACTICS trial received sorafenib treatment for a much longer period than in previous trials, with a median of 38.7 weeks and 17.0 to 21.0 weeks, respectively. This new TACE-specific endpoint and protocol should be validated in future TACE combination trials.

Another approach being investigated is the combination of immune checkpoint inhibitors with TACE (Table 3). Locoregional therapies, including TACE, activate the host immune system by promoting local inflammation and triggering the release of tumor antigens [99]. When tumor antigens are released by TACE, subsequent administration of immune checkpoint inhibitors can prevent intrahepatic micrometastases, which are typically undetectable, and are the main cause of recurrence [100]. One prospective study demonstrated that ablative therapies, such as RFA and TACE, induced a peripheral immune response and enhanced the efficacy of tremelimumab in patients with advanced-stage HCC [101]. The combination of TACE and tremelimumab afforded favorable outcomes, with a partial response rate of 26% and OS of 12.3 months. At present, a phase III trial of combination therapy with TACE plus durvalumab and/or bevacizumab (the EMERALD-1 trial) is ongoing (NCT03937830).

## 7. Models to Predict Prognosis after TACE

Since intermediate-stage HCC consists of a heterogeneous group of patients, not all intermediate HCC patients can benefit from TACE [102]. Patients with intermediate-stage HCC present with a broad range of tumor burdens, tumor biology, liver function, and comorbidities. Adequate patient selection for TACE is crucial to maximize the therapeutic effect. Therefore, in addition to the staging systems for HCC, other selection criteria have been developed to predict treatment response after TACE to aid in decision making.

### 7.1. New Prognostic Models

The heterogeneity of intermediate-stage HCC and the common use of TACE outside of the recommended guidelines have inspired the evolution of scoring systems to predict patient survival. Various prognostic models have been developed to predict survival after TACE, including the hepatic arterial embolization prognostic (HAP) score [103], the selection for transarterial chemoembolization treatment (STATE) score [104], the modified HAP score [105], the modified HAP-II score [106], the SANCOR model [107], the prognostic nomogram [108], and the modified HAP-III score [109]. However, research on a simple prognostic model based on routinely available parameters is still required. Recently, several other new prognostic models have been developed to predict survival after TACE. Lee et al. developed a new prognostic model consisting of the albumin-bilirubin (ALBI) grade, Up-to-11 criteria [20] and the α-fetoprotein (AFP) level (ALBI-TAE model) [110]. The ALBI-TAE model was superior to other prognostic models in both the training and validation datasets, as well as in the overall cohort, and this can be applied to select patients who may benefit the most from TACE.

As a new prognostic model, Wang et al. developed a six-and-twelve score that can predict individual outcomes with favorable discrimination, and with the sum of tumor size and number ≤6, >6 but ≤12, and >12 The score identified three prognostic levels presenting significantly different median survival values of 49.1 months, 32.0 months, and 15.8 months, respectively [111]. The six-and-twelve score may prove an easy-to-use tool to stratify the recommended TACE candidates and predict individual survival with favorable performance and discrimination. Recently, Han et al. presented TACE-specific models that permit accurate individualized patient survival prediction [112]. They built both a pre-TACE model (“Pre-TACE-Predict”) and a post-TACE model (“Post-TACE-Predict”), which included the first mRECIST response, in addition to the baseline features. This TACE-specific model, based on the routinely available clinical features and responses after the first TACE, permitted patient classification into four different risk categories, wherein the median OS ranged from seven months to more than four years. The model and its online calculator enable patient-level prognostication, which may help physicians rationalize the application of TACE by inhibiting intervention in patients with a predicted poor prognosis.

### 7.2. Neutrophil-to-lymphocyte Ratio as a Prognostic Biomarker

Serum components are the most promising biomarkers for HCC surveillance, as they ensure easy performance and rapid measurement. A high neutrophil-lymphocyte ratio (NLR) has been associated with poor survival in HCC patients undergoing TACE [113,114,115,116]. As a marker of systemic inflammation, NLR is associated with cancer progression, metastasis, and prognosis in various cancers. Neutrophils are closely associated with tumor cell proliferation and survival, as well as tumor angiogenesis, metastasis, and disruption of the acquired immune system [117]. Meanwhile, lymphocytes are key players in cancer immune surveillance, suppressing tumor progression [118]. Therefore, decreased lymphocyte counts have been associated with impaired anti-tumor immune responses, thus enabling tumor progression and metastasis [119]. NLR with other combined factors, such as the platelet to lymphocyte ratio (PLR) [113], aspartate aminotransferase-to-alanine aminotransferase ratio [120], or C-reactive protein to albumin ratio [121], can promote survival prediction after TACE. In addition, the prognostic score, including NLR, was remarkable compared with the prior scores [116]. Baseline NLR and its dynamic changes during therapy can predict OS in HCC patients treated with TACE. However, one limitation of NLR is that the baseline NLR cutoff values are different in each study [122]. Different NLRs applied to each center result in various data and are confusing for clinical use. Studies including large cohorts of patients are required to establish the most appropriate NLR cutoff value, which provides good sensitivity and specificity.

### 7.3. Machine Learning (Radiomics) and Deep Learning

Machine learning refers to a subcategory of artificial intelligence research [123]. Deep learning refers to a subfield of machine learning that depends on multiple processing layers to learn generalizable representations of data with higher levels of abstraction [124,125]. Among the many machine learning techniques, radiomics, which was presented in 2012 by Lambin et al. [126], has awakened interest. Radiomics is defined as the conversion of images to higher-dimensional data, and the subsequent mining of these data to allow improved decision support [127]. It is characterized by the extraction of quantitative imaging features from conventional imaging modalities using computer-based algorithms and the correlation of these features with relevant clinical endpoints, such as pathology, therapeutic response, and survival [128]. Radiomics studies can generally be divided into five phases: data selection, segmentation, feature extraction, exploratory analysis, and modeling [129]. Quantitative radiomics analysis and models have been shown to accurately predict outcomes in patients undergoing TACE [130,131,132], as well as CT, MRI, and PET CT (Table 4). Meng et al. selected the six most predictive radiomics features from the training cohort. Of these six features, two were based on arterial phase imaging from the tumor volume of interest (VOI) and peritumoral VOI, respectively, and the remaining four features were from the tumor VOI on portal venous phase imaging. The radiomic signature and tumor number (< 4 vs. ≥ 4) were incorporated into a combined radiomics-clinic (CRC) model to predict OS in patients with HCC undergoing TACE. They determined that the CT radiomics signature represents an independent biomarker of survival in patients with HCC undergoing TACE, and the CRC model displayed improved predictive performance [131]. In a study investigating pretreatment PET imaging in patients with unresectable HCC undergoing ^90^Y radioembolization, a whole liver radiomics score including both the tumor and background liver was predictive of both progression-free survival (PFS) and OS [133]. NLR and PLR were recently correlated with the radiomic features extracted from pretreatment contrast-enhanced MRI and with tumor response and PFS after DEB-TACE [134]. In this study, high NLR and PLR were associated with non-nodular tumor growth, measured as low tumor sphericity. This combined prediction with immunologic biomarkers and radiomics will provide a new paradigm for personalized application. Nevertheless, radiomics is a contemporary method that can be further improved in order to help overcome the limitations inherent to complex computer-dependent models, and in particular, the lack of standardization of image acquisition, e.g., the reconstruction kernel or section thickness, which can hide important underlying biological textural features [127]. To date, the use of deep learning in liver imaging and radiology is very limited, and its performance has not been validated. There is still much to learn from and about deep learning and its potential applications [135].

## 8. Conclusions

We reviewed the latest data on the use of TACE in treating HCC patients from various perspectives. Typically, there are two types of TACE: cTACE and DEB-TACE. Physicians can use either technique, with the knowledge that there is a higher risk of hepatic artery and biliary injuries and a relatively lower risk of post-procedural pain after DEB-TACE than after cTACE. TACE can be used for early stage HCC, as well as for intermediate-stage disease if other curative modalities are not feasible, and can be adopted as a neoadjuvant treatment before LT. In addition, TACE can be considered when treating selected patients with segmental PVTT and preserved liver function. Repeated TACE can be determined based on the ART and ABCR scores. It is important to recognize TACE failure/refractoriness and provide patients with more personalized therapeutic regimens. Combination therapy of TACE with RFA and RT is well established. TACE plus RFA is favorable for large HCCs, whereas TACE plus RT is specialized for HCC with vascular invasion. Research on the application of TACE with systemic therapy is still actively ongoing; in particular, the combination of TACE with immunotherapy is expected. Recently, new prognostic models (ALBI-TAE model, six-and-twelve score, TACE specific model), neutrophil-lymphocyte ratio, and radiomics and deep learning have been developed to predict survival after TACE.

## Figures and Tables

**Table 1:** Parameters used in ART and ABCR scores.

	ART	ABCR
BCLC stage		A: 0 point B: 2 points C: 3 points
AFP		≥ 200 ng/mL: 1 point
Child-Pugh score	1-point increase: 1.5 point ≥ 2-point increase: 3 points	≥ 2-point increase: 2 points
Radiologic tumor response	No: 1 point	Yes: −3 points
AST	> 25% increase: 4 points	
Score of ineffectiveness	ART score ≥ 2.5	ABCR score ≥ 4

ART, Assessment for Retreatment of TACE; ABCR, α-fetoprotein, BCLC, Child-Pugh, and Response; BCLC, Barcelona Clinic of Liver Cancer; AFP, α-fetoprotein; AST. Aspartate transaminase.

**Table 2:** Definition of TACE failure/refractoriness by the Japanese Society of Hepatology.

Item	Definition
Intrahepatic lesion	i. Two or more consecutive insufficient responses of the treated tumor (viable lesion > 50%) even after changing the chemotherapeutic agents and/or reanalysis of the feeding artery. The response evaluation is based on CT or MRI at 1–3 months after adequately performed selective TACE.
	ii. Two or more consecutive increases of tumor number even after having changed the chemotherapeutic agents and/ or reanalysis of the feeding artery. The response evaluation is based on CT or MRI at 1–3 months after adequately performed selective TACE.
Tumor markers	Continuous elevation of tumor markers after TACE even though transient decrease is observed.
Vascular invasion	Appearance of vascular invasion
Metastasis	Appearance of extrahepatic spread

TACE, transarterial chemoembolization; CT, computed tomography; MRI, magnetic resonance imaging.

**Table 3:** Selected studies using the combination of TACE and systemic therapy.

Combination Modality with TACE	Trial Identifier	Study Duration	Treatment	Number	Primary Endpoint and Results
Anti-angiogenic therapy	SPACE, NCT00855218	2009.03–2013.02	Sorafenib with DEB-TACE vs. placebo with DEB-TACE	307	Sorafenib plus DEB-TACE did not improve TTP compared with DEB-TACE alone [93]
Anti-angiogenic therapy	TACE 2, ISRCTN93375053	2010.11–2015.12	Sorafenib with DEB-TACE vs. placebo with DEB-TACE	313	Sorafenib plus DEB-TACE did not improve PFS compared with DEB-TACE alone [94]
Anti-angiogenic therapy	BRISK-TA, NCT00908752	2009.07–2012.09	Brivanib after TACE vs. placebo after TACE	502	Brivanib as adjuvant therapy to TACE did not improve OS [95]
Anti-angiogenic therapy	ORIENTAL, NCT01465464	2010.12–2014.06	Orantinib with TACE vs. placebo with TACE	889	Orantinib combined with TACE did not improve OS [96]
Anti-angiogenic therapy	TACTICS, NCT01217034	2010.10–2018.03	Sorafenib with TACE vs. TACE alone	228	Median PFS was significantly longer in the TACE plus sorafenib than in the TACE alone group [98]
ICI	NCT01853618	2013.03–2017.06	Tremelimumab with RFA or TACE	32	Partial response rate, 26%; OS, 12.3 months [101]
ICI	NCT03638141	2019.06–2020.11	Durvalumab in combination with tremelimumab after DEB-TACE	30	ORR, not available (ongoing)
ICI	NCT03143270	2017.04–2022.04	Nivolumab with DEB-TACE	14	Number of participants with treatment-related adverse events (ongoing)
ICI	IMMUTACE, NCT03572582	2018.06–2023.06	Nivolumab with TACE	49	ORR, not available (ongoing)
ICI	PETAL, NCT03397654	2018.01–2020.12	Pembrolizumab after TACE	26	Number of participants with treatment-related adverse events (ongoing)
ICI	NCT03592706	2009.12–2021.08	Immune killer cells and TACE vs. TACE	60	Change of tumor size, PFS, not available (ongoing)
ICI and anti-angiogenic therapy	NCT03937830	2020.09–2022.12	Durvalumab, bevacizumab and DEB-TACE	22	PFS, not available (ongoing)

TACE, transarterial chemoembolization; ICI, immune checkpoint inhibitor; DEB, drug-eluting beads; RFA, radiofrequency ablation; TTP, time-to-progression; OS, overall survival; PFS, progression-free survival; ORR, objective response rate.

**Table 4:** Summary of studies that applied radiomics in predicting progression and survival after transarterial chemoembolization.

Author	Treatment	Imaging Modality	Extraction Software	Segmentation	Readers	Model	Validation	Number (Training/Validationor Test Sets)	Primary Endpoint	Results
Kim et al. [130]	TACE	CECT	Matlab	Manual ROI	1	Y	N	NA	OS and PFS	Combining clinical and radiomic features better predicted survival
Meng et al. [131]	TACE	CECT	Pyradiomics	Manual VOI	2	Y	I	108/54	OS	CT radiomics signature represented an independent biomarker of OS
Sun et al. [132]	TACE	MP-MRI	Pyradiomics	Manual VOI	3	Y	I	67/17	PD	Preoperative MP-MRI has the potential to predict the outcome of TACE

TACE, transarterial chemoembolization; CECT, contrast-enhanced computed tomography; ROI, region of interest; Y, yes; N, no; NA, not available; OS, overall survival; PFS, progression-free survival; VOI, volume of interest; I, internal; MP-MRI, multiparametric magnetic resonance imaging; PD, progressive disease; ^90^Y-TARE, transarterial radioembolization using yttrium-90; ^18^F-FDG PET/CT, positron emission tomography with 2-deoxy-2-[fluorine-18] fluoro-D-glucose integrated with computed tomography; MITK, Medical Imaging Interaction Toolkit.
